# Toll-Like Receptor Ligands and Interferon-γ Synergize for Induction of Antitumor M1 Macrophages

**DOI:** 10.3389/fimmu.2017.01383

**Published:** 2017-10-26

**Authors:** Elisabeth Müller, Panagiotis F. Christopoulos, Sanjib Halder, Anna Lunde, Kahsai Beraki, Martin Speth, Inger Øynebråten, Alexandre Corthay

**Affiliations:** ^1^Tumor Immunology Lab, Department of Pathology, Rikshospitalet, Oslo University Hospital, University of Oslo, Oslo, Norway; ^2^Department of Biosciences, University of Oslo, Oslo, Norway

**Keywords:** macrophages, tumoricidal, toll-like receptors, interferon-γ, cancer, nitric oxide, immunotherapy

## Abstract

Tumor-associated macrophages may either promote or suppress tumor growth depending on their activation status. Interferon-γ (IFN-γ) has been identified as a key factor for inducing tumoricidal M1 phenotype in macrophages. However, it remains unclear whether IFN-γ is sufficient or if additional stimuli are required. Here, we tested IFN-γ and a panel of toll-like receptor (TLR) agonists for the ability to activate murine macrophages toward a tumoricidal M1 phenotype. The following TLR ligands were used: TLR1/TLR2 agonist Pam3CSK4, TLR2/TLR6 agonist lipotechoic acid, TLR3 agonist poly(I:C), TLR4 agonist lipopolysaccharide (LPS), TLR5 agonist flagellin, TLR7 agonist CL264, and TLR9 agonist CpG. We used an *in vitro* growth inhibition assay to measure both cytotoxic and cytostatic activity of mouse macrophages against Lewis lung carcinoma (LLC) and MOPC315 plasmacytoma tumor cells. Production of nitric oxide (NO) and cytokines by activated macrophages was quantified. We found that IFN-γ alone was not able to render macrophages tumoricidal. Similarly, macrophage activation with single TLR agonists was inefficient. In sharp contrast, IFN-γ was shown to synergize with TLR agonists for induction of macrophage tumoricidal activity and production of both NO and pro-inflammatory cytokines (TNF-α, IL-12p40, and IL-12p70). Furthermore, IFN-γ was shown to suppress macrophage IL-10 secretion induced by TLR agonists. NO production was necessary for macrophage tumoricidal activity. We conclude that two signals from the microenvironment are required for optimal induction of antitumor M1 macrophage phenotype. Combination treatment with IFN-γ and TLR agonists may offer new avenues for macrophage-based cancer immunotherapy.

## Introduction

Macrophages are multifunctional cells whose activities are triggered in response to stimuli from the microenvironment. The stroma of solid tumors contains tumor-associated macrophages (TAMs) which may either suppress or promote tumor development depending on their activation phenotype (1, 2). According to a widely used nomenclature, macrophages with antitumor or killing activity are called M1 while tumor-promoting or healing macrophages are named M2 or M2-like (3, 4). Because TAMs are commonly assumed to have a tumor-promoting phenotype, research in the field has mainly focused on detrimental aspects of macrophages in tumors (5) and therapeutic strategies were designed accordingly for the depletion of TAMs (6). However, it has also been reported that TAMs may be rendered tumoricidal upon activation by tumor-specific Th1 cells (7). Furthermore, several recent reports revealed the potential of re-programming TAMs toward a tumoricidal M1 phenotype rather than depleting them (8–10). Therefore, it is of therapeutic importance to clarify the molecular requirements for activation of macrophages toward an antitumor M1 phenotype.

Antitumor M1-polarized macrophages are characterized by their direct cytostatic and cytotoxic effect on tumor cells, secretion of pro-inflammatory cytokines, and stimulation of T cell immunity (7, 11, 12). The ability of macrophages to kill tumor cells *in vitro* was reported already in 1970 (13), and it was shown that supernatant of spleen cells from tumor-immunized mice contained a factor that could render macrophages tumoricidal *in vitro* (14). Investigations into the cooperation of lymphoid cells and macrophages led to the identification of interferon-γ (IFN-γ), previously known as macrophage-activating factor (MAF), as a major agent responsible for regulating macrophage tumoricidal activity (15, 16). Bacterial endotoxin [lipopolysaccharide (LPS)] and viral RNA were also reported to render macrophages cytotoxic to tumor cells (17). Later studies suggested that IFN-γ may not be sufficient to render macrophages tumoricidal and that a second signal from the microenvironment was required (18, 19). Dead bacteria or purified LPS were shown to provide such a second signal to render macrophages tumoricidal in combination with IFN-γ (20–22). Still, many current reviews refer to IFN-γ as the major inducer of tumoricidal M1 macrophages or do not make a distinction between the phenotype resulting from activation with IFN-γ alone, LPS alone or both factors (23, 24). A popular view is that activation of M1/M2 macrophage phenotypes depend on cytokines from adaptive immune cells (such as IFN-γ from Th1 cells or IL-4 from Th2 cells), rather than signals from innate receptors such as toll-like receptors (TLRs) (25). There is confusion regarding the current annotation of macrophage phenotype and the M1/M2 classification has been criticized (24, 26). Recent studies investigating macrophage activation do not describe the direct tumoricidal activity of macrophages, but rather focus on production of cytokines, nitric oxide (NO) and reactive oxygen species, and changes in gene expression or surface markers (27, 28). As a result, it remains unclear whether IFN-γ is sufficient or if additional stimuli such as LPS are required for induction of tumoricidal M1 macrophages.

Lipopolysaccharide binds to TLR4, a member of the TLR family of receptors which recognize pathogen- and damage-associated molecular pattern molecules. These receptors signal through adaptor molecules and downstream mediators to modulate gene transcription and induce a pro-inflammatory response. The great potency of LPS in stimulating immune responses has led to clinical trials investigating the use of LPS against cancer. Unfortunately, severe side effects have been reported and therapeutic use of LPS against cancer has so far not been approved (29). However, TLR4 agonists different from LPS as well as agonists of other TLRs have been investigated for their potential use in cancer therapy, either as vaccine adjuvants or immune modulators (30). Several TLR agonists have been shown to activate macrophages similarly to LPS, inducing cytokine production, upregulation of antigen-presentation and co-stimulatory molecules, and induction of the enzyme inducible NO synthase (iNOS) with resulting NO production (31, 32). Viral double stranded RNA, an agonist of TLR3, was shown to induce tumoricidal activity in macrophages already in the 1970s (17), and a synthetic analog, poly(I:C), was also efficient (33). Other TLR agonists that have shown potency for induction of antitumor M1 macrophages includes lipotechoic acid (LTA) (34), gardiquimod (35), R848 (36), and CpG (37). However, none of these studies investigated the role of IFN-γ in potentiating the effect of the TLR agonists despite accumulating evidence for the strong synergistic effect of this cytokine on TLR signaling. Furthermore, the experimental setup, including the source of macrophages, the functional assay used, and the activation regimen with single or combination treatment varied greatly between these studies. This makes it difficult to conclude on the background of the current literature as to which TLRs are effective in inducing tumoricidal M1 macrophages and whether IFN-γ is important for such activation.

This prompted us to systematically test a range of TLR agonists in functional assays for M1 polarization of mouse macrophages, including tumoricidal activity, and to test the synergistic effect of IFN-γ when combined with LPS or other TLR agonists. We found that several TLR agonists could induce macrophage-mediated tumor cell growth inhibition, but only when combined with IFN-γ. IFN-γ synergized with all TLR agonists for NO production and secretion of pro-inflammatory and Th1-polarizing (IL-12p70) cytokines. We conclude that optimal activation of antitumor M1 macrophages require two signals, which can be provided by a combination of IFN-γ and TLR agonists. Seven TLR agonists were shown to be effective and thereby emerge as potential therapeutic agents for cancer immunotherapy based on targeting of TAMs.

## Materials and Methods

### Mice

C57BL/6NRj mice from Janvier Labs (Le Genest-Saint-Isle, France) were bred at the Department of Comparative Medicine, Oslo University Hospital, Rikshospitalet (Oslo, Norway) in specific pathogen free conditions. The study was approved by the Norwegian National Committee for Animal Experiments. All experiments were performed in accordance with the institutional guidelines and regulations, including EU directive 2010/63/EU.

### Cell Lines

MOPC315 is a mineral-oil induced plasmacytoma cell line which was derived from a BALB/c mouse and was purchased from the American Type Culture Collection (ATCC, Manassas, VA, USA) (38). Lewis lung carcinoma (LLC, also called LLC1) is a cell line originating from a spontaneous lung carcinoma in a C57BL/6 mouse and was obtained from CLS Cell Lines Service (Eppelheim, Germany) (39). L929 is a fibroblast-like cell line originating from connective tissue of a C3H/An mouse and was obtained from ATCC (40). J774.A1 is a macrophage-like cell line originating from the ascites of a BALB/c mouse with reticulum cell sarcoma and was kindly given by Anders Ø. Gammelsrud at the Norwegian Veterinary Institute (Oslo, Norway) (41). All of the above described cell lines were negative for mycoplasma infection as tested by use of MycoSensor PCR Assay kit (#302109, Agilent, Santa Clara, CA, USA).

### Generation of Bone Marrow Derived Macrophages

Confluent L929 cells were cultured in RPMI 1640 medium (#61870044, Thermo Fisher Scientific, Waltham, MA, USA) containing 10% fetal bovine serum (FBS, #BCHRS0405, Biochrom GmbH, Berlin, Germany) for 10 days before the medium was centrifuged, filtered and stored at −20°C. Such L929 cell-conditioned medium contains macrophage colony-stimulating factor and was used for induction of macrophage differentiation according to standard protocols (42). Femurs and tibiae of the hind legs from 8- to 12-week-old male and female C57BL/6NRj mice were harvested and flushed with RPMI 1640 medium containing 10% FBS under sterile conditions. The isolated bone marrow was passed through a cell strainer with 70-µm pores (#CLS431751-50EA, Sigma-Aldrich, St. Louis, MO, USA) and cultured in 15 mm non-tissue culture treated dishes (#734-2359, VWR, Radnos, PA, USA) in RPMI 1640 medium containing 30% L929-derived conditioned medium. The bone marrow cells were cultured for 5 days, after which non-adherent, cells were washed off using phosphate buffered saline (PBS, # D8662, Sigma-Aldrich) and the adherent macrophages were cultured for 1 more day. Macrophages were harvested by incubation for 30 min at 4°C with cold PBS without CaCl_2_ and MgCl_2_ (#D8537, Sigma-Aldrich). Macrophages were then flushed off the plate, collected and kept frozen in aliquots at −150°C in FBS with 10% dimethyl sulfoxide (#0231-500 ml, VWR) for future experiments. The purity of the cells was 99% when analyzed by flow cytometry using the macrophage markers CD11b (#101219, BioLegend, San Diego, CA, USA) and F4/80 (#123116, BioLegend), and the cells were then referred to as bone marrow derived macrophages (BMDMs).

### Tumor Cell Growth Inhibition Assay

Bone marrow derived macrophages were thawed and cultured for 3 days in non-tissue culture treated dishes (VWR) in RPMI 1640 medium containing 10% FBS and 10% L929-derived conditioned medium. The BMDMs were harvested by scraping, incubated for 2 h at 37°C with 10 mg/ml mitomycin C (#M4287, Sigma-Aldrich) to inhibit proliferation, and then washed thoroughly. Next, the BMDMs were resuspended in medium and seeded out in triplicates in flat bottom 96 well plates (#734-1793, Costar, Washington, DC, USA) at 3 cell densities: 6 × 10^4^, 3 × 10^4^, and 3 × 10^3^ cells/well in a final volume of 200 μl/well. After 24 h of incubation, the medium was replaced with medium containing various stimuli, see section below, and incubated for another 24 h. Then, half of the cell supernatants (100 µl) were removed and used for quantification of NO_2_^−^. Target cell suspensions, consisting of 5,000 cells/well of MOPC315 or 3,000 cells/well of LLC cells were added, resulting in varying ratios of effector to target cells. After 24 h of co-culture, cells were pulsed with [^3^H]thymidine (#MT6032, Hartmann Analytic, Braunschweig, Germany) and harvested 18 h later by a freeze and thaw cycle. The amount of radiolabeled DNA was measured on a 1450 MicroBeta Trilux Microplate Scintillation counter (Perkin Elmer, Waltham, MA, USA). The same assay was performed using the cell line J774.A1 as effector cells instead of BMDMs. Mitomycin C (10 mg/ml) was also used to block proliferation of J774.A1 cells before they were seeded out in 96 well plates in triplicates at a density of 1 × 10^5^ cells/well in a final volume of 200 μl/well.

For the purpose of statistical analysis of pooled data from several experiments, the percentage of remaining cancer cell growth was calculated by dividing counts per minute (cpm) values from the macrophage-LLC co-cultures at the 20:1 ratio with the cpm values of the corresponding wells with LLCs alone using the following equation:

$$\frac{{\text{cpm}}_{20:1}}{{\text{cpm}}_{\text{LLC alone}}}\times 100=\text{\% growth remaining}.$$

### TLR Agonists and Cytokines

The following TLR agonists were used: TLR1/TLR2 agonist Pam3CSK4 (Pam3, #tlrl-pms, InvivoGen, San Diego, CA, USA); TLR3 agonist poly(I:C) of high molecular weight type (#tlrl-pic, InvivoGen); TLR4 agonist LPS from *E. coli* (#L4391, Sigma-Aldrich); TLR5 agonist flagellin (FLA) from *S. typhimurium*, ultrapure type (#tlrl-epstfla-5, InvivoGen); TLR2/TLR6 agonist LTA from *S. aureus* (#L2515, Sigma-Aldrich); TLR7 agonist CL264 (#tlrl-c264e-5, InvivoGen); and TLR9 agonist CpG, class C ODN 2395 (#tlrl-2395-1, InvivoGen). The TLR agonists were used alone or in combination with mouse recombinant IFN-γ (#315-05, Peprotech, Rocky Hill, NJ, USA) at a 40 ng/ml concentration.

### Quantification of Nitrite by the Griess Test

Supernatants were centrifuged at 410 g to remove cellular debris and immediately assayed for nitrite as a measure for the amount of NO that was produced. 50 µl of macrophage supernatant was added to 50 µl of Griess reagent A consisting of distilled water with 1% sulphanilamide (#S9251, Sigma-Aldrich) and 5% phosphoric acid (#W290017, Sigma-Aldrich). The mixture was incubated in the dark for 10 min. Next, 50 µl of Griess reagent B consisting of 0.1% *N*-(1-napthyl) ethylenediamine (#N9125, Sigma-Aldrich) in distilled water was added and the absorbance at 540 nm was measured with a microplate reader (BioTek Instruments, Winooski, VT, USA). Serial dilution of NaNO_2_ served to create a standard curve of nitrite in the range of 1.56–100 µM.

### iNOS Inhibition and NO Donor

*S*-Methylisothiourea hemisulfate salt (SMT, #M84445, Sigma-Aldrich) is a potent inhibitor of iNOS (43) which was used to block the production of NO by activated macrophages. Diethylenetriamine/NO adduct (DETA/NO) (#D185, Sigma-Aldrich) was used to produce controlled release of NO in solution.

### Cytokine Quantification by Luminex Technology

Supernatants harvested from macrophages that had been stimulated with TLR agonists and/or IFN-γ for 24 h were centrifuged to remove cellular debris and stored at −80°C for maximum 1 week and assayed for cytokines. The cytokine concentrations were determined by multiplex bead assays, Bio-Plex Pro Mouse cytokine singleplex sets for TNF-α (#171-G5023M), IL-12p40 (#171-G5010M), IL-12p70 (#171-G5011M), monokine-induced by IFN-γ (MIG) (#171-G6005M), and IL-10 (#171-G5009M) from Bio-Rad Laboratories (Hercules, CA, USA) according to the manufacturer’s instructions. Samples in duplicates were analyzed, using a Bio-Plex MAGPIX Multiplex Reader and Bio-Plex Manager 6.1 software (Bio-Rad Laboratories).

### Statistical Analysis

Multiple groups were compared by using one-way ANOVA followed by a *post hoc* Tukey test for multiple comparisons and *p* values of less than 0.05 were considered statistically significant (*p-value < 0.05, **p-value < 0.01, ***p-value < 0.001). Statistical analysis, including column statistics, was performed using GraphPad Prism 7.02 software.

## Results

### LPS and IFN-γ Synergize to Activate Macrophages to Inhibit Tumor Cell Growth

Table 1 shows an overview of the literature on induction of tumoricidal activity of macrophages by various TLR agonists. The most widely used agonist, LPS, has shown effect in a number of studies that utilized different functional assays, macrophages and target cells. LPS has been used alone, in combination with MAF/IFN-γ, other TLR agonists, agonistic anti-CD40 antibodies, or TGF-β inhibition. However, basic questions regarding the induction of tumoricidal activity in normal macrophages remain to be answered. Many of the studies from the 1970s and 1980s lacked reliably pure (LPS free) reagents or macrophages, and more recent articles typically lack functional assays for tumoricidal activity.

**Table 1:** Literature on induction of macrophage tumoricidal activity by TLR agonists.

TLR agonist	Functional assay used	Effector cells	Target cells	Conclusion	Ref
Lipopolysaccharide (LPS), lipid A (TLR4), RNA, and poly(I:C) (TLR3)	GI by manual counting of cells	PM from DBA/2 or CBA mice	L5178Y	All agents induced tumoricidal activity in macrophages	(17)
LPS	GI by cell number, cell death by release of thymidine	PM from C3H/HeN, C3H/HeJ mice	3T12	LPS induced tumoricidal activity, and MAF acted synergistically	(20)
LPS	Cell death by thymidine labeling	Human macrophages from PBMCs	SK-BR-3 and HT-29	LPS induce tumoricidal activity, and MAF acted synergistically	(44)
Lipotechoic acid (LTA) (TLR2/6), lipomannan (TLR2)	Cell death by release of thymidine	Bone marrow derived macrophages from DA rats	P-815 and DA tumor cells	Some LTAs induced strong tumoricidal activity, other LTAs and lipomannan less	(34)
LPS, CpG (TLR9)	GI by thymidine labeling and cell death by flow cytometry	PM from C3h/HeJ, CB17 SCID/beige or C57BL/6 mice	L5178Y, B16, RENCA, M21, NXS2, OVCAR	CpG and LPS combined or either factor combined with *in vivo* anti-CD40 ligation induced tumoricidal activity	(45)
LPS, BCG	Cell death by chrome release assay	PM from C3H/HeN mice	MBT-2	Both LPS and BCG induced tumoricidal activity	(46)
LPS, CpG	GI by thymidine labeling and cell death by flow cytometry	PM from C57BL/6 mice	B16, L5178Y and NXS2	*In vivo* stimulation with CpG induced tumoricidal activity *in vitro*, which was improved by adding LPS	(47)
Poly(I:C)	Cell death by chrome release assay	Tumor-associated macrophages (TAMs) from C57BL/6 mice	3LL Lewis	*In vivo* poly(I:C) induced *in vitro* tumoricidal activity	(33)
LPS, CpG	GI by thymidine labeling	PM from C57BL/6 mice	B16	LPS synergized with *in vivo* stimulation with anti-CD40 and CpG to induce *in vitro* tumoricidal activity	(48)
LPS, gardiquimod (TLR7)	Cell death by assay for luciferase	CD11b^+^ CD11c^−^ TAMs from C57/BL6 mice	MN/MCA1	TGF-β inhibition combined with TLR4 or TLR7 ligation induced tumoricidal activity	(35)
R848 (TLR7/8), Pam3 TLR(1/2)	Cell death by flow cytometry	mMDCs from human PBMCs	A549	R848 induced tumoricidal activity, but not Pam3	(36)

*GI, growth inhibition; PM, peritoneal macrophages; ds, double stranded; MAF, macrophage-activating factor (interferon-γ); PBMCs, peripheral blood mononuclear cells; BCG, Bacillus Calmette–Guérin; TGF-β, tumor necrosis factor beta; iNOS, inducible nitric oxide synthase; mMDCs, monocytic myeloid-derived suppressor cells*.

**Figure 1:**
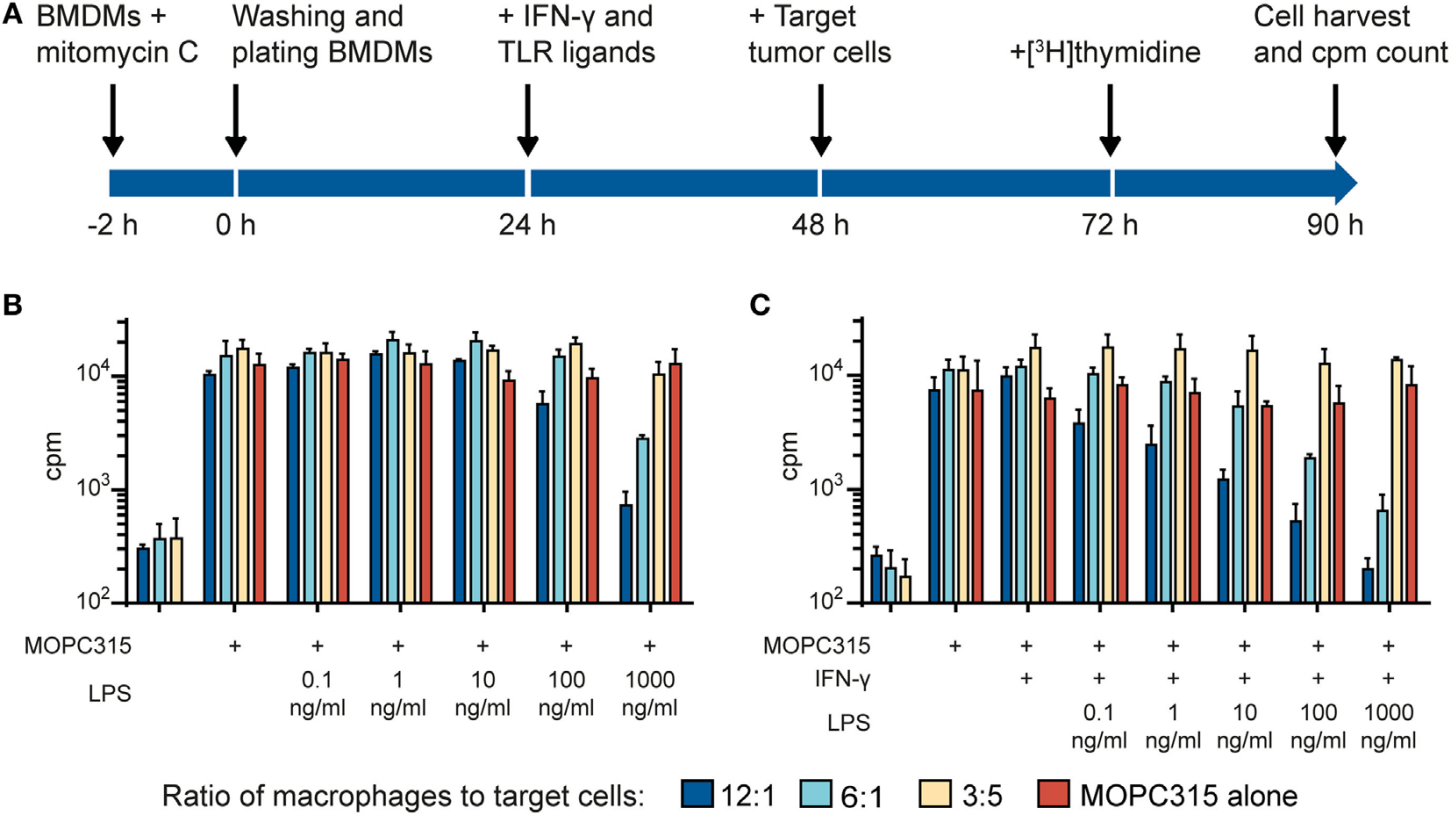
Lipopolysaccharide (LPS) and IFN-γ synergize to induce tumoricidal activity in macrophages. **(A)** Time-line for the growth inhibition assay used for measuring macrophage cytotoxic and cytostatic activity toward tumor cells. **(B)** Mitomycin C-treated bone marrow derived macrophages (BMDMs) were stimulated for 24 h with various LPS concentrations before addition of 5,000 MOPC315 tumor cells/well, resulting in the indicated macrophage to target cell ratios. The growth of the tumor cells was quantified by measuring incorporation of radiolabeled thymidine and is shown on the *y*-axis as mean counts per minute (cpm) values of triplicates ± SD. The three columns to the left show proliferation of BMDMs alone (tumor cells were not added). **(C)** BMDMs were stimulated with IFN-γ (40 ng/ml) in combination with different concentrations of LPS for 24 h before target cells were added. Radiolabeled thymidine incorporation in growing cells is shown on the *y*-axis as mean cpm values of triplicates ± SD. The three columns on the left show proliferation of BMDMs alone. **(B,C)** All experiments were performed three times and representative experiments are shown.

We used an *in vitro* growth inhibition assay (7, 13) in order to measure both the cytotoxic and cytostatic activity of macrophages toward tumor cells (Figure 1A). Macrophages were first treated with the DNA crosslinker mitomycin C to block cell division and thereby avoid that macrophage growth could interfere with the detection of tumor cell growth inhibition. Thereafter, macrophages were cultivated in the presence or absence of IFN-γ and TLR agonists for 24 h, before tumor cells were added and co-cultured for 42 h. Radiolabeled thymidine was used to detect tumor cell growth and was added to the co-cultures 18 h before cell harvest. As inhibition of tumor cell growth is known to depend on the number and density of macrophages, we seeded out macrophages in three different cell concentrations while the number of tumor cells remained constant within an experiment. The resulting ratio of macrophages to tumor cells, i.e., ratio of effector to target cells varied from 20:1 to 1:1 in various experiments.

In the first set of experiments, we investigated the effect of the classical macrophage activators IFN-γ and LPS. We used C57BL/6-derived BMDM as a source of normal mouse macrophages and the MOPC315 plasmacytoma as target tumor cells. When used alone, a high concentration (1,000 ng/ml) of LPS was required to activate BMDMs for inhibition of MOPC315 cell growth (Figure 1B). The potency of LPS was greatly improved when the macrophages were stimulated with LPS in combination with IFN-γ (Figure 1C) in accordance with previous reports (20, 22). Notably, macrophage stimulation with IFN-γ alone had no inhibitory effect on tumor cell growth (Figure 1C). Taken together, the experiments showed that activation with both LPS and IFN-γ was required for optimal induction of tumoricidal activity in macrophages. LPS alone could induce tumoricidal M1 macrophages, but only at high concentrations, while stimulation with IFN-γ alone had no effect.

### Several TLR Agonists Other than LPS Synergize with IFN-γ for Rendering Macrophages Tumoricidal

**Figure 2:**
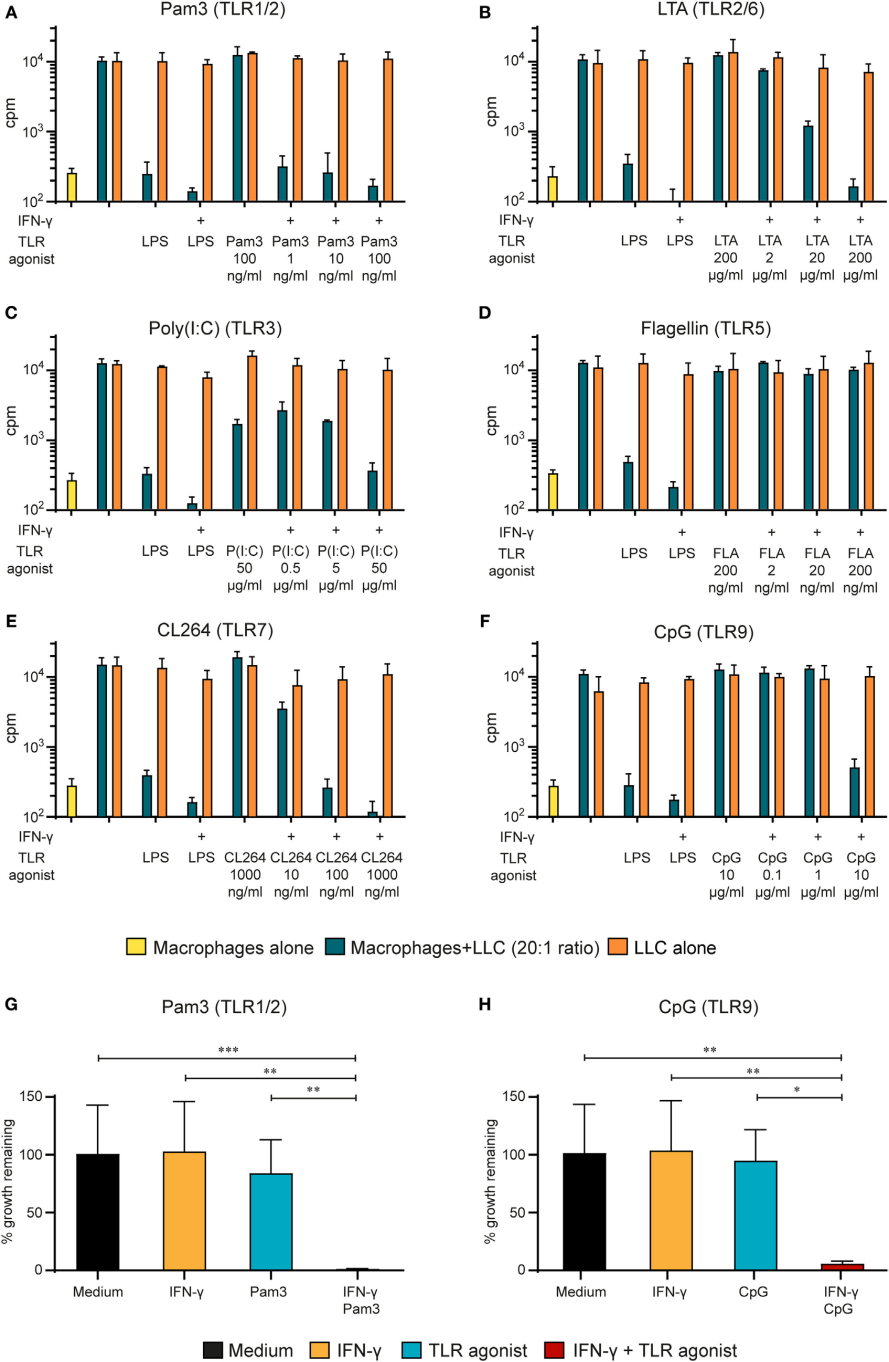
Synergy between IFN-γ and several TLR agonists for M1 macrophage activation. **(A–F)** Mitomycin C-treated bone marrow derived macrophages (BMDMs) (6 × 10^4^ cells/well) were stimulated for 24 h with several TLR agonists at various concentrations in the presence or absence of IFN-γ (40 ng/ml) before addition of 3,000 LLC tumor cells/well, resulting in a 20:1 macrophage to target cell ratio. lipopolysaccharide (LPS) (1 µg/ml) + IFN-γ (40 ng/ml) was used as a positive control for macrophage activation. Radiolabeled thymidine incorporation in growing cells is shown on the *y*-axis as mean cpm values of triplicates ± SD. The first column on the left show proliferation of BMDMs alone. The following TLR agonists were tested at the indicated concentrations: **(A)** TLR1/2 agonist Pam3CSK4; **(B)** TLR2/6 agonist lipotechoic acid (LTA); **(C)** TLR3 agonist Poly(I:C); **(D)** TLR5 agonist Flagellin; **(E)** TLR7 agonist CL264; and **(F)** TLR9 agonist CpG. All experiments were performed three times and representative experiments are shown. **(G,H)** Statistical analysis of the pooled results from 5 **(G)** and 4 **(H)** growth inhibition assays performed as described above with the indicated TLR agonists. *y*-axis show % remaining growth calculated by dividing cpm_20:1_ by cpm_LLC_ alone and multiplying with 100. *p*-values from multiple comparison test using one-way ANOVA is displayed as follows: *p-value < 0.05, **p-value < 0.01, ***p-value < 0.001.

To explore the potential of other natural or synthetic TLR agonists for inducing tumoricidal M1 macrophage phenotype, we tested a panel of agonists targeting different TLRs. In these experiments, the Lewis lung carcinoma (LLC) cell line was used as target cell line anticipating that macrophage-mediated tumor cell growth inhibition was not restricted to a single cell line. The target tumor cells were added to BMDMs activated by the following TLR agonists; TLR1/2 agonist Pam3, TLR2/6 agonist LTA, TLR3 agonist poly(I:C), TLR5 agonist flagellin, TLR7 agonist CL264, and TLR9 agonist CpG (Figures 2A–F). Pam3 was very potent at stimulating the BMDMs and it resulted in strong growth inhibition of LLC cells, even at concentrations as low as 1 ng/ml, but only when it was used together with IFN-γ (Figure 2A). Similarly, IFN-γ in combination with LTA (Figure 2B), CL264 (Figure 2E), and CpG (Figure 2F) induced tumor cell growth inhibition by BMDMs. Stimulation of BMDMs with poly(I:C) resulted in growth inhibition both alone and together with IFN-γ. Similar to LPS, the effect of poly(I:C) was potentiated by IFN-γ (Figure 2C). Stimulation of BMDM with flagellin yielded no growth inhibition (Figure 2D). Statistical analysis was performed for two TLR agonists (Pam3 and CpG) by pooling data from experimental repeats. Because baseline cpm values varied between experiments, the percentage of growth remaining was used in the comparisons. The analysis revealed a statistically significant stronger growth inhibition when macrophages were activated by two signals (TLR agonist and IFN-γ) compared to one signal only (Figures 2G,H). Thus, induction of tumoricidal M1 macrophages can be achieved through stimulation of the TLRs 1/2, 2/6, 3, 4, 7, or 9 when combined with IFN-γ. Stimulation of TLR 3 and 4 has some effect alone at high ligand concentrations. The only TLR agonist tested that did not activate BMDMs was flagellin (TLR5).

### A Macrophage Cell Line also Inhibits Tumor Cell Growth Following Stimulation with TLR Agonists and IFN-γ

**Figure 3:**
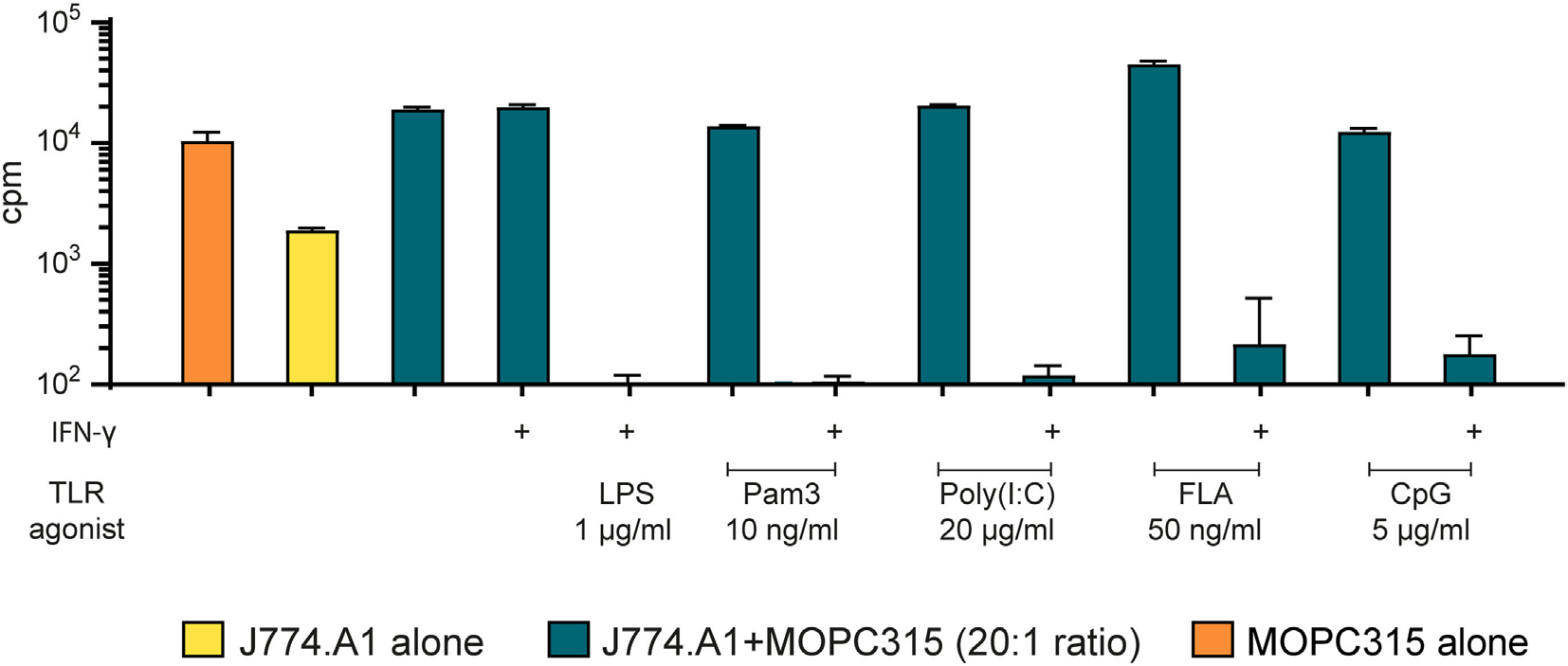
The macrophage cell line J774.A1 inhibits tumor cell growth in a similar manner as bone marrow derived macrophages after two-signal activation. Growth inhibition assays. Mitomycin C-treated J774.A1 cells (1 × 10^5^ cells/well) were stimulated with TLR agonists as indicated in the presence or absence of IFN-γ (40 ng/ml) for 18 h before addition of 5,000 MOPC315 tumor cells/well, resulting in a 20:1 effector to target cell ratio. Radiolabeled thymidine incorporation in growing cells is shown on the *y*-axis as mean cpm values of triplicates ± SD. The first column on the left shows proliferation of target cells alone and the second column shows proliferation of effector cells alone. This experiment was performed three times and a representative experiment is shown.

To investigate whether our findings were of a more general value rather than being specific to BMDMs, we tested an immortalized murine macrophage cell line, J774A.1, in the growth inhibition assay. Mitomycin C was used to block J774A.1 cell growth, before stimulation with TLR agonists alone or in combination with IFN-γ. When activated by LPS and IFN-γ, the macrophage cell line induced very strong growth inhibition of MOPC315 cells (Figure 3). These results were consistent with the growth inhibition mediated by BMDMs (Figure 2). We observed similar effect of co-stimulation with IFN-γ and the agonists Pam3 and CpG for the J774.A1 cell line and the BMDMs, whereas the effect of poly(I:C) combined with IFN-γ was stronger for the cell line. IFN-γ and flagellin also successfully stimulated J774A.1 to inhibit growth. Thus, murine macrophages, either primary cells or a cell line, could be activated toward a tumoricidal M1 phenotype by stimulation with IFN-γ and a second signal. Several TLR agonists could provide this second signal.

### Tumor Cell Growth Inhibition by Activated Macrophages Is Mediated by NO

**Figure 4:**
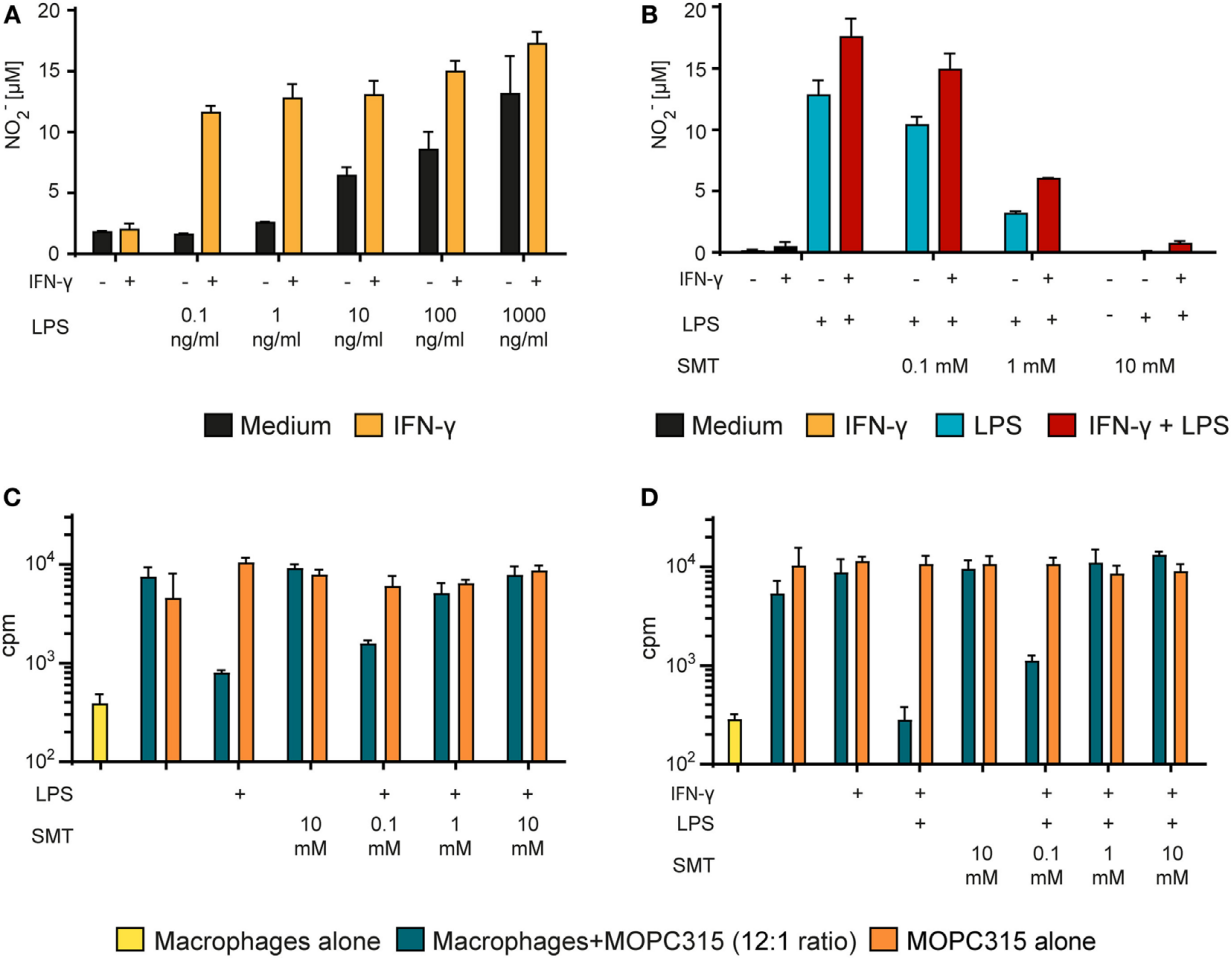
Tumor cell growth inhibition by activated macrophages is mediated by NO. **(A)** Bone marrow derived macrophages (BMDMs) (6 × 10^4^ cells/well) were stimulated with different concentrations of lipopolysaccharide (LPS) alone or in combination with IFN-γ (40 ng/ml) for 24 h. The Griess assay was used to measure NO in the supernatants indirectly as nitrite (NO_2_^−^). NO_2_^−^ levels (μM) are presented as mean values of triplicates ± SD. **(B)** BMDMs (6 × 10^4^ cells/well) were incubated with various concentrations of the inducible NO synthase inhibitor SMT (S-Methylisothiourea hemisulfate salt) and stimulated with LPS (1 µg/ml) alone or in combination with IFN-γ (40 ng/ml) for 24 h. NO_2_^−^ concentration (μM) in the supernatants was measured using the Griess assay and presented as mean values of triplicates ± SD. **(C,D)** Growth inhibition assay. Mitomycin C-treated BMDMs (6 × 10^4^ cells/well) were stimulated for 24 h with LPS alone (1 µg/ml) **(C)** or with LPS (1 µg/ml) + IFN-γ (40 ng/ml) **(D)** and treated with various concentrations of SMT before addition of 5,000 MOPC315 tumor cells/well, resulting in a 12:1 macrophage to target cell ratio. Radiolabeled thymidine incorporation in growing cells is shown on the *y*-axis as mean cpm values of triplicates ± SD. The first column on the left show proliferation of BMDMs alone. **(A–D)** All experiments were performed three times and representative experiments are shown.

Production of the cytotoxic free radical NO is considered a hallmark of M1-polarized pro-inflammatory macrophages (49). NO was shown to be crucial for macrophage-mediated defense against bacteria during normal immune responses (50) and has been reported to be important for the killing of tumor cells *in vitro* (51, 52). Due to the extremely short half-life of NO, we quantified it indirectly using the Griess assay. This assay is based on the Griess diazotization reaction of the NO metabolite nitrite (NO_2_^−^) which forms a colored azo compound that can be quantified with a spectrophotometer. We analyzed the supernatant of BMDMs during the growth inhibition assay just before tumor cells were added. Macrophage activation with LPS alone for 24 h resulted in a concentration-dependent NO production (Figure 4A). Stimulation with LPS in combination with IFN-γ greatly potentiated the effect and yielded more than 10 µM NO_2_^−^ already at the lowest concentration of LPS that was tested (0.1 ng/ml) (Figure 4A). At 1,000 ng/ml of LPS, there was no clear additive effect of co-stimulation with IFN-γ, and the NO_2_^−^ production seemed to reach a maximum level around 15 µM. These results, where stimulation with IFN-γ greatly improved the effect of LPS, are in accordance with previous studies on NO induction (53). These data also support our finding in the growth inhibition assay, showing that stimulation with two signals is required for optimal induction of M1 macrophage phenotype, defined either by tumoricidal activity or NO production.

To investigate the importance of NO in macrophage-mediated tumor cell growth inhibition, we used the iNOS-specific inhibitor SMT to block NO production (43). SMT completely blocked NO production by activated BMDMs when used at 10 mM concentration, whereas 1 mM only partly hindered NO production (Figure 4B). When tested in the growth inhibition assay, 1 mM SMT was sufficient to abolish the growth inhibition induced both by LPS alone (Figure 4C) and by LPS in combination with IFN-γ (Figure 4D). These data strongly suggest that macrophages mediate growth inhibition of tumor cells through a NO-dependent mechanism.

### Cell-free NO Is Cytotoxic at a High Concentration

**Figure 5:**
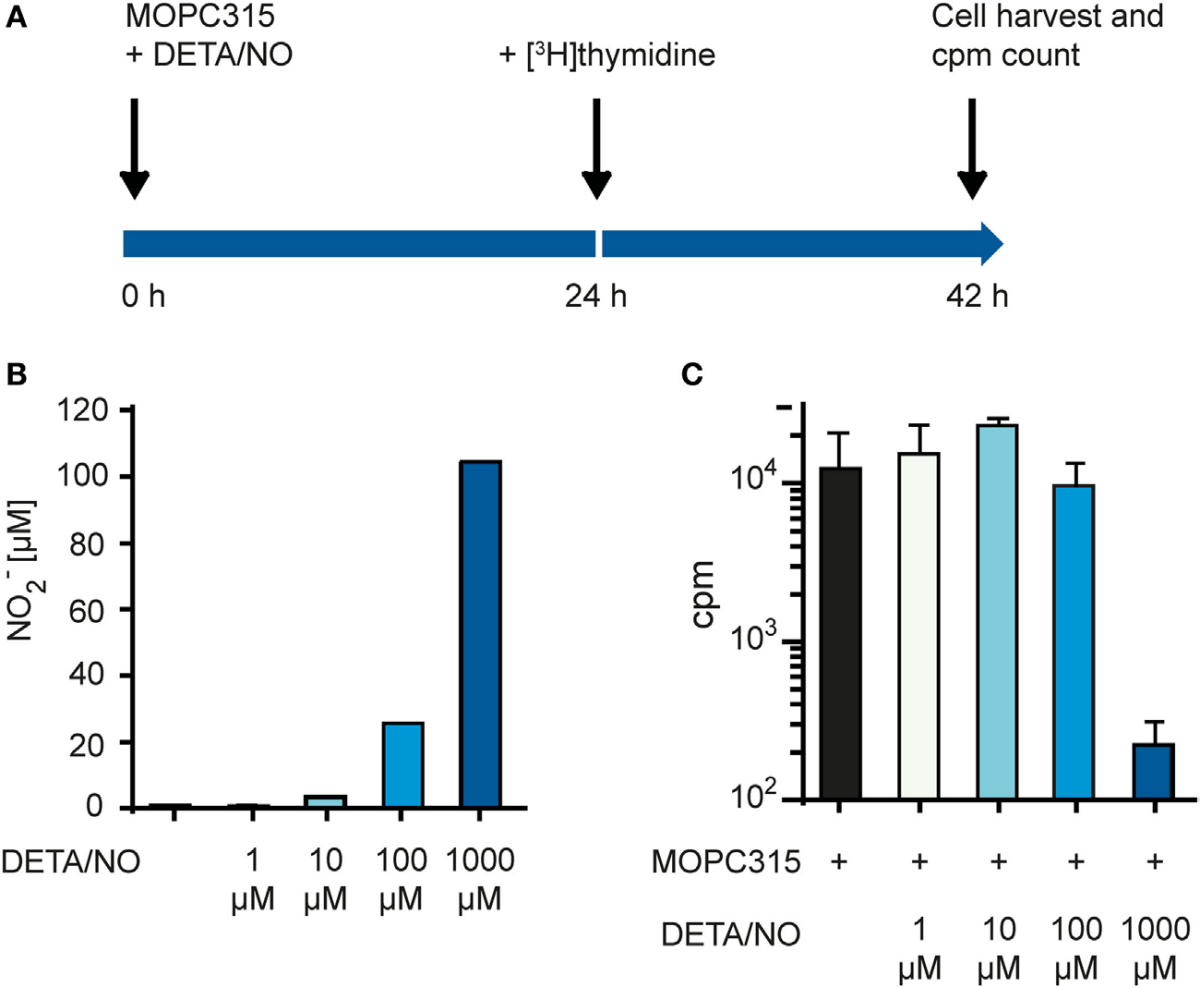
Tumor cell proliferation is inhibited by high concentrations of NO. **(A)** Time-line for the growth inhibition assay used for measuring direct cytotoxic and cytostatic activity of NO released from diethylenetriamine/nitric oxide adduct (DETA/NO) toward tumor cells. **(B)** Varying concentrations of DETA/NO in media was analyzed for NO_2_^−^ using the Griess assay. The *y*-axis shows the μM concentration of NO_2_^−^ measured. **(C)** Growth inhibition assay. MOPC315 cells were cultured in varying concentrations of DETA/NO for 42 h before analysis. Radiolabeled thymidine incorporation in growing cells is shown on the *y*-axis as mean cpm values of triplicates ± SD. All experiments were performed three times and representative experiments are shown.

To test whether we could recreate the growth inhibitory effect of NO without the presence of macrophages, we used the chemical compound diethylenetriamine/NO adduct (DETA/NO), which functions as an NO donor and releases NO to the medium. We set up a modified growth inhibition assay where tumor cells were exposed to DETA/NO in the absence of macrophages (Figure 5A). DETA/NO was dissolved in cell culture medium and used immediately. Just before adding the DETA/NO solution to the MOPC315 target cells, the amount of NO released in the medium was quantified indirectly by measuring NO_2_^−^ for each concentration of DETA/NO used (Figure 5B). MOPC315 growth was then quantified by measuring incorporation of radiolabeled thymidine as for the standard growth inhibition assay (Figure 5C). We found that inhibition of growth was obtained only at the highest tested DETA/NO concentration, i.e., 1 mM, which corresponds to a NO_2_^−^ concentration of around 100 µM (Figures 5A,B). These data confirm that our target tumor cells are sensitive to NO in a concentration-dependent way and are consistent with a key role of NO secretion for macrophage tumoricidal activity.

### TLR Agonists Mediate Tumor Cell Growth Inhibition *via* Production of NO

**Figure 6:**
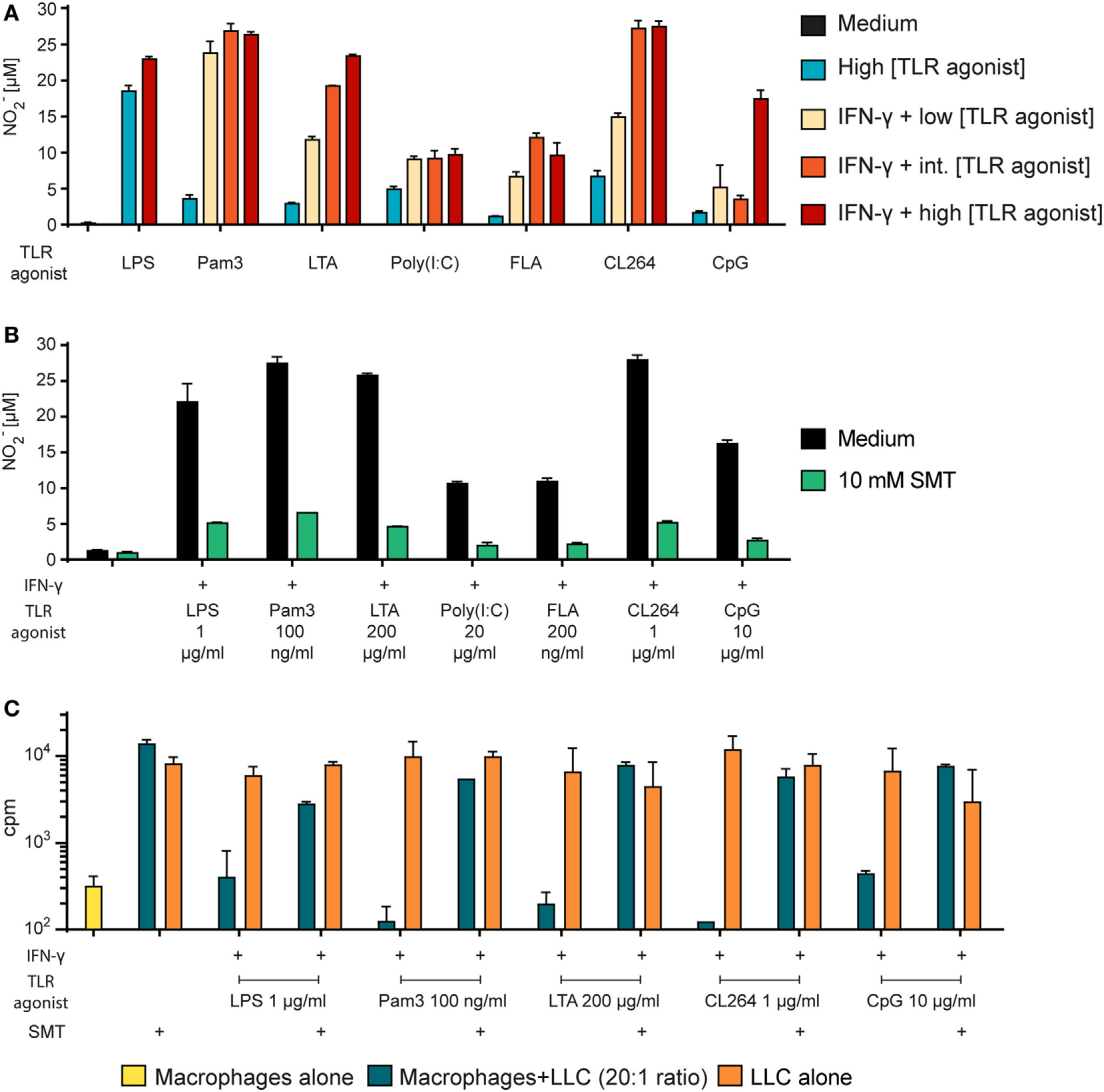
Tumor cell growth inhibition by macrophages activated by any TLR agonist requires NO. **(A)** Bone marrow derived macrophages (BMDMs) (6 × 10^4^ cells/well) were stimulated with TLR agonists alone or in combination with IFN-γ (40 ng/ml) as indicated for 24 h. NO_2_^−^ concentration (μM) in the supernatants was measured using the Griess assay and presented as mean values of triplicates ± SD. Each of the TLR agonists were tested at three concentrations (low/intermediate (int)/high): Pam3 (1/10/100 ng/ml); lipotechoic acid (LTA) (2/20/200 μg/ml); poly(I:C) (0.5/5/50 μg/ml); flagellin (FLA) (2/20/200 ng/ml); CL264 (10/100/1,000 ng/ml); CpG (0.1/1/10 μg/ml). **(B)** BMDMs (6 × 10^4^ cells/well) were incubated in the absence or presence of the inducible NO synthase inhibitor s-methylisothiourea hemisulfate salt (SMT) (10 mM) and stimulated with TLR agonists as indicated and IFN-γ (40 ng/ml) for 24 h. NO_2_^−^ concentration (μM) in the supernatants was measured using the Griess assay and presented as mean values of triplicates ± SD. **(C)** Growth inhibition assay. Mitomycin C-treated BMDMs (6 × 10^4^ cells/well) were incubated in the absence or presence of SMT (10 mM) and IFN-γ (40 ng/ml) in combination with TLR agonists as indicated for 24 h before addition of 3,000 LLC tumor cells/well, resulting in a 20:1 macrophage to target cell ratio. The growth of the tumor cells was quantified by measuring incorporation of radiolabeled thymidine and is shown on the *y*-axis as mean cpm values of triplicates ± SD. The first column to the left show control wells with BMDMs alone (no tumor cells). **(A–C)** All experiments were performed three times and representative experiments are shown.

To examine whether TLR agonists other than LPS also induce NO production, we stimulated BMDMs with TLR agonists both alone and in combination with IFN-γ and measured the levels of NO_2_^−^ in the supernatants (Figure 6A). For each TLR agonist, three concentrations were chosen and arbitrary defined as low, intermediate, and high. We found that all TLR agonists synergized with IFN-γ for induction of NO production, as the NO_2_^−^ levels were 2- to 10-fold higher when BMDMs were activated with TLR agonists in combination with IFN-γ compared with TLR agonists alone. IFN-γ together with a low concentration of the TLR agonists still yielded more NO than single activation with TLR agonist at a 100-fold higher concentration (Figure 6A). Thus, several TLR agonists can replace LPS as an activating signal for macrophage NO production.

Next, we wanted to verify that the inhibitor SMT could inhibit the NO production induced by any TLR agonist. Measurements by the Griess assay revealed that SMT reduced the levels of NO_2_^−^ in the BMDM cultures stimulated with TLR agonists in combination with IFN-γ (Figure 6B). Furthermore, we observed that the growth inhibition induced after co-stimulation with IFN-γ and LPS or any other tested TLR agonist was abolished when the iNOS inhibitor was present (Figure 6C). Therefore, *in vitro* tumor cell growth inhibition after macrophage activation with any TLR agonist appears to depend on NO production. Table 2 shows a summary of the effect of all tested TLR agonists in combination with IFN-γ. There was a strong, but incomplete correlation between induction of NO production and tumor cell growth inhibition.

**Table 2:** Summary of TLR-mediated activation of macrophages in synergy with IFN-γ.

Activation signal^a^		Bone marrow derived macrophages		J774.A1	
		
Agonist	TLR	GIA^b^	NO^c^	GIA	NO
Lipopolysaccharide	TLR4	++	++	++	++
Pam3	TLR1/2	++	++	++	+
Lipotechoic acid	TLR2/6	++	++	ND	ND
Poly(I:C)	TLR3	+	+	++	+
Flagellin	TLR5	−	+	++	++
CL264	TLR7	++	++	ND	ND
CpG	TLR9	++	++	++	+

*^a^Given in combination with IFN-γ*.

*^b^Tumor cell growth inhibition assay (GIA): +, some inhibition; ++, strong inhibition; −, none; ND, not determined*.

*^c^Nitric oxide (NO) production. +, some; ++, strong; −, none; ND, not determined*.

### IFN-γ and TLR Agonists Synergize for Production of Pro-inflammatory and Th1-Polarizing Cytokines by BMDMs

**Figure 7:**
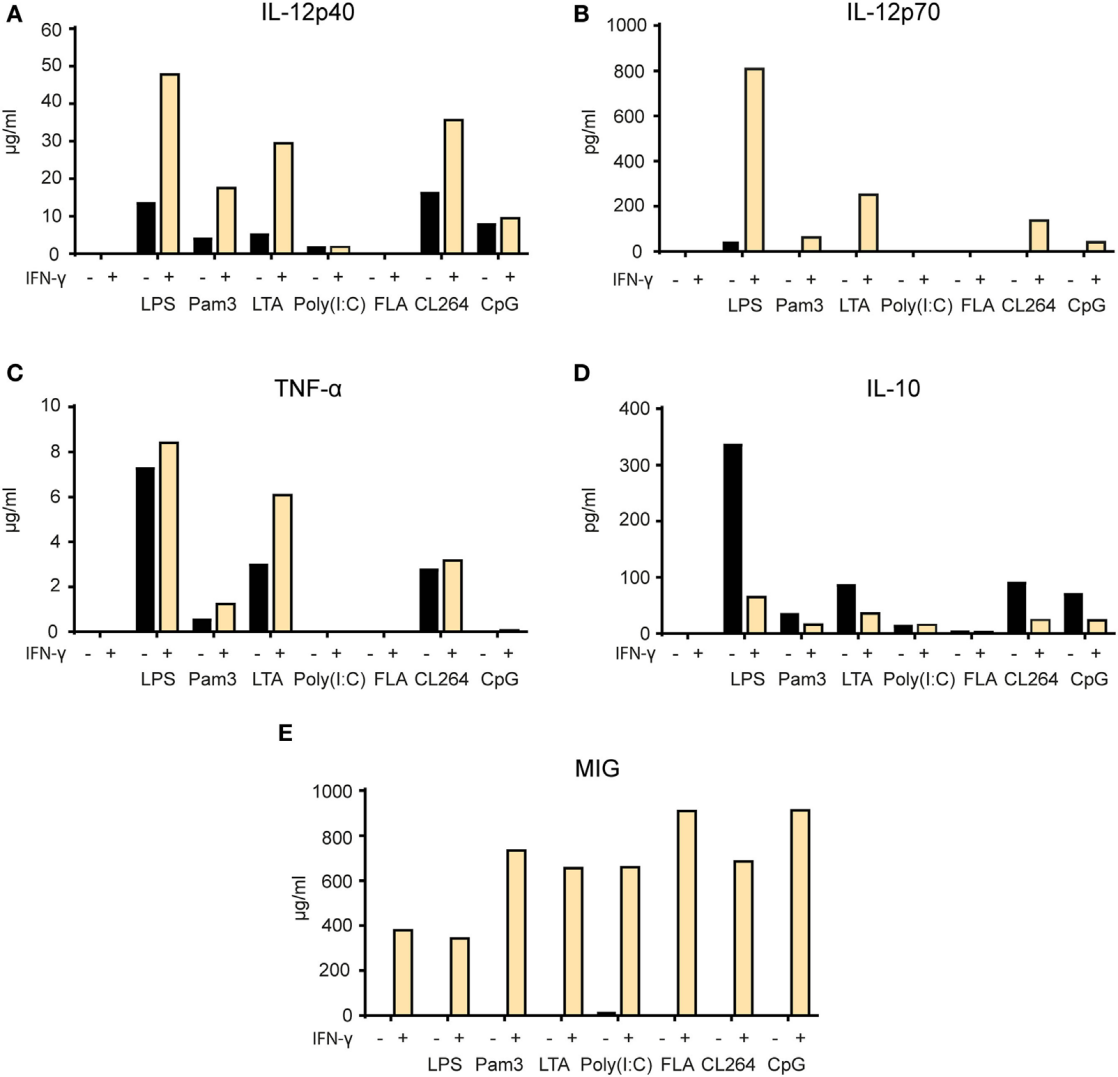
Synergy between IFN-γ and TLR agonists for induction of pro-inflammatory cytokine secretion by macrophages. **(A–E)** Mitomycin C-treated bone marrow derived macrophages (2.4 × 10^4^ cells/well) were stimulated for 24 h with the following TLR agonists in the presence or absence of IFN-γ (40 ng/ml): lipopolysaccharide (LPS) (1 µg/ml), Pam3 (100 ng/ml), lipotechoic acid (LTA) (200 µg/ml), poly(I:C) (50 µg/ml), flagellin (200 ng/ml), CL264 (1 µg/ml), and CpG (10 µg/ml). Cell supernatants were analyzed by Luminex technology and the cytokine content is shown on the *y*-axis as mean pg/ml or ng/ml values of duplicates. The following cytokines were measured: **(A)** IL-12p40, **(B)** IL-12p70, **(C)** TNF-α, **(D)** IL-10, and **(E)** monokine-induced by IFN-γ (MIG). All experiments were performed three times and representative experiments are shown.

In the next set of experiments, we wanted to examine whether release of particular cytokines was affected by single versus two signal activation of macrophages. We measured the levels of the pro-inflammatory cytokines TNF-α and IL-12p40, the Th1-polarizing cytokine IL-12p70, the anti-inflammatory cytokine IL-10, and the chemokine MIG/CXCL9 in the supernatant of BMDMs stimulated for 24 h (Figure 7). There was a clear synergistic effect of IFN-γ and most TLR agonists on the secretion of TNF-α, IL-12p40, and IL-12p70. Activation with TLR agonists alone resulted in relatively low to medium cytokine levels which increased in response to LPS, Pam3, LTA, and CL264 when IFN-γ was added (Figures 7A–C). IL-10 production was induced by TLR agonists alone with LPS giving the strongest response. Strikingly, IL-10 production induced by TLR triggering was reduced in the presence of IFN-γ (Figure 7D). Exceptions were poly(I:C) and flagellin, which resulted in no or very low secretion of any cytokine both when used alone and in combination with IFN-γ. Untreated BMDMs produced no cytokines. BMDMs activated with IFN-γ alone secreted no cytokines except for MIG/CXCL9 as expected for this IFN-γ-inducable chemokine. The chemokine MIG/CXCL9 was strongly induced by IFN-γ alone and the levels were further increased upon combined activation with all TLR agonists, except LPS (Figure 7E). Thus, IFN-γ and TLR agonists synergize to make macrophages produce high levels of pro-inflammatory and Th1-polarizing cytokines (TNF-α, IL-12p40, and IL-12p70) and low levels of IL-10.

## Discussion

In this paper, we show that activation with two molecular signals from the microenvironment is required for efficient induction of M1 phenotype in murine macrophages as defined by tumoricidal activity, NO production, and secretion of pro-inflammatory and Th1-polarizing cytokines. We evaluated first two classical macrophage stimulators, namely LPS and IFN-γ. We found that IFN-γ greatly potentiates the effect of LPS, resulting in strong tumoricidal activity at low LPS concentrations, whereas no tumoricidal activity was induced by IFN-γ alone. A similar synergistic effect of LPS and IFN-γ on induction of tumoricidal macrophages was shown previously by several investigators in the 1970s and 1980s (16, 18, 19, 21, 22). However, the interpretation of many of these early studies is problematic due to variability in the source of macrophages and potentially impure or LPS-contaminated IFN-γ (previously called MAF) preparations. Peritoneal macrophages were used in most of the studies, and often peptone or thioglycollate was injected into the peritoneum to increase the yield of macrophages. These compounds may themselves give an inflammatory stimulus to the macrophages (54). Moreover, peritoneal macrophages may be contaminated by other cell types (55), and this is not accounted for in all studies. The literature also contains reports on induction of tumoricidal M1 macrophages by single activation with IFN-γ or LPS (56, 57), and most recent reviews make no distinction between the macrophage phenotypes resulting from activation with IFN-γ, LPS, or both. Due to the potential of M1 macrophages for immunotherapy for cancer, a clarification of which signals are required for an optimal induction of these cells was needed. In pilot studies, we used peritoneal macrophages, but considerable variability between experiments was observed (data not shown). Therefore, we decided to use BMDM generated by standard protocols as source of normal mouse macrophages. Using BMDMs as effector cells, we could clearly show that IFN-γ alone is ineffective at activating macrophages to a tumoricidal M1 phenotype. LPS had some effect alone, but only when it was used in high concentrations, indicating that M1 activation by LPS alone is sub-optimal. When macrophages were activated with IFN-γ in combination with LPS, a potent tumoricidal phenotype was obtained even with the use of very low LPS concentrations. Thus, our data confirm earlier *in vitro* studies with LPS and IFN-γ that revealed that two signals are required for inducing a tumoricidal M1 macrophage phenotype. This is also in line with our previous findings from an *in vivo* model of myeloma, where IFN-γ was required, but not sufficient to explain the cytotoxic effect of TAMs, indicating the involvement of another signal (7, 11).

Based on our findings of the synergistic effect of IFN-γ and the TLR4 agonist LPS, we wanted to investigate whether stimulation with LPS could be replaced by triggering any other TLR. Some TLR agonists have previously been reported to be able to induce tumoricidal M1 macrophages, but the TLR ligands were mostly used in combination with other agents such as TGF-β inhibitors or CD40 agonists rather than IFN-γ (see Table 1). Synergistic effects of several TLR agonists and IFN-γ on macrophage expression of cytokines and NO production has been described (58, 59), but to the best of our knowledge the only TLR ligands that have been shown to synergize with IFN-γ for induction of tumoricidal functions of macrophages are LPS and poly(I:C) (60). We therefore set up a panel of agonists covering most of the well-described TLRs in mice. We found that all TLR agonists synergized with IFN-γ to induce a tumoricidal M1 macrophage phenotype. Flagellin, a TLR5 agonist, combined with IFN-γ did not induce any tumor cell growth inhibition by BMDMs, but it activated the macrophage cell line J774.A1. This could be explained by various factors such as lower TLR5 receptor expression by BMDMs compared to J774.A1. Importantly, all TLR agonists, with the exception of LPS and poly(I:C), had no effect when used alone, but induced potent macrophage-mediated tumor cell growth inhibition when combined with IFN-γ. This may explain the lack of reports on induction of tumoricidal M1 macrophages by other TLR agonists, as previous studies have not included IFN-γ in the activation protocol (Table 1). Several recent studies revealed the therapeutic potential of activating TAMs toward an antitumor M1 phenotype, resulting in macrophage-mediated tumor immune surveillance with tumor regression *in vivo* (8–10). These proof-of-principle reports support the potential application of our findings in the development of novel macrophage-targeted cancer therapies by combining IFN-γ with TLR agonists.

Our experiments demonstrated that the presence of NO was necessary for cancer cell growth inhibition by macrophages, which is consistent with recent studies reporting the importance of NO in macrophage-mediated antitumor effects (61, 62). NO was found to be the main mediator of the tumoricidal effect of activated macrophages in a number of studies from the 1980s (51, 52, 63), but some reports also indicate the existence of iNOS-independent mechanisms (64). Inhibition of iNOS in activated macrophages resulted in a concentration-dependent abrogation of both NO production and tumor cell growth inhibition. Production of NO by BMDMs correlated with tumor cell growth inhibition, but could not be used as a predictive surrogate marker for tumoricidal activity (Table 2). This finding has consequences for the interpretation of previous studies as well as the planning of future studies aimed at inducing tumoricidal M1 macrophages. M1 macrophages were originally defined as having a killer phenotype with a characteristic shift in l-arginine metabolism into NO production, as opposed to healing M2 macrophages which use l-arginine to generate l-ornithine and urea (3, 65). Consequently, induction of iNOS, the enzyme responsible for production of NO by activated macrophages, has been established as a hallmark of tumoricidal M1 macrophages (66). We would argue that the widespread use of iNOS-expression or NO production by macrophages as a surrogate marker of tumoricidal M1 macrophages should be replaced or accompanied by functional assays that directly measure the tumoricidal activity of macrophages.

We observed a synergistic effect of IFN-γ and TLR agonists on the induction of the pro-inflammatory cytokines TNF-α and IL-12p40, and the Th1-polarizing cytokine IL-12p70 (also called IL-12p75), while the angiostatic chemokine MIG (or CXCL9) was induced by IFN-γ alone. We have previously reported in mouse models for myeloma and lymphoma that the secretion of these cytokines was associated with successful immunity against cancer (11). Furthermore, production of TNF-α and IL-12 cytokines plays important roles in macrophage-mediated immune responses to pathogens and cancer (67), and the observation that TLR agonists and IFN-γ synergize for this function fits well with previous studies (68, 69). Interestingly, the results for the anti-inflammatory cytokine IL-10 were different, as IFN-γ reduced the induction of IL-10 seen by TLR activation alone. The ability of IFN-γ to inhibit IL-10 production has been previously described and suggested as a potential mechanism underlying the synergistic effect of IFN-γ on TLR-mediated macrophage activation (70). IL-10 is induced at a low level upon TLR activation and mediates a negative feedback loop involving induction of STAT3 (71, 72). IFN-γ was shown to inhibit IL-10 production by increasing the activity glycogen synthase kinase 3β (GSK-3β), a negative regulator of AP-1 and CREB signaling (70). GSK-3β mediated the synergistic activity of IFN-γ on increasing NF-κB activity, NO production and IL-6 secretion in TLR-activated macrophages (73, 74). So far, GSK-3β has been shown to be a key regulator of TLR2 and TLR4 signaling, and potentially also TLR3 (75). However, more studies are required to clarify the role of GSK-3β in the synergistic effect of IFN-γ and other TLRs, as well as whether this regulatory pathway can explain why a combination of IFN-γ and TLR agonists are required for optimal induction of tumoricidal M1 activity in macrophages.

Our data confirm previous findings showing that LPS and poly(I:C) may induce some macrophage-mediated tumor cell growth inhibition in the absence of IFN-γ (17). At first glance, this contradicts our conclusion that M1 macrophage polarization requires two signals. However, it has been reported that LPS and poly(I:C) might in fact act by combining TLR signaling with autocrine type I interferon signaling (76). Torres and Johnson demonstrated that both LPS and poly(I:C) induced secretion of IFN-α/β and that the tumoricidal activity induced by LPS or poly(I:C) could be abrogated by neutralizing antibodies against IFN-α/β, but not against IFN-γ (76). Adding IFN-γ to poly(I:C)-activated macrophages after IFN-α/β-blocking could rescue the tumoricidal activity. Furthermore, Pace et al. observed that both IFN-α and β could synergize with LPS or heat killed *Listeria monocytogenes* for the induction of tumoricidal activity, however less potently than IFN-γ (77). After the discovery of the receptors that recognize LPS and poly(I:C), TLR4 and TLR3 respectively, and the signaling pathways involved, it has become clear that these two TLRs share the ability to signal *via* a TRIF-dependent pathway, resulting in activation of IRF3 and induction of type I interferons (78, 79). The other main signaling pathway used by TLRs depends on MyD88 and results in activation of NFκB rather than IRF3 (80). TLR4 is the only TLR that is able to activate both pathways, and this has been suggested to explain the powerful effect of LPS on macrophage activation. Synergistic effects on cytokine production and T cell stimulation from combined activation of macrophages with MyD88-dependent and TRIF-dependent TLR agonists have previously been described (81), and may provide a novel way of inducing tumoricidal M1 macrophages. Thus, the two-signal model for induction of tumoricidal M1 macrophages might be extended to encompass interferon-α/β/γ-signaling and signaling through a large range of TLRs. Such insight on 2-signal requirement should be valuable for the development of future macrophage-targeted cancer therapies.

Our data suggest a general mechanism of TLR and IFN-γ-mediated signaling that synergizes for induction of antitumor M1 macrophage phenotype. The striking functional similarities between different TLR agonists suggest that differential TLR expression between mouse and human macrophages might not represent a major problem for therapy development, since multiple TLR agonists may potentially be used. It has been shown that monocyte-derived human macrophages could inhibit tumor cell growth *in vitro* upon combined activation with LPS and IFN-γ (44), suggesting that the rules for induction of M1 macrophage phenotype may be conserved across these two species. Another important issue that will need clarification is whether TLR activation in combination with IFN-γ will be sufficient to induce M1 phenotype in TAMs which are considered to be polarized differently in M2 or M2-like modus. Such repolarization has been reported using several activation protocols, including miRNA (82). Interestingly, a TLR7 agonist was shown to be effective at reversing the pro-tumor phenotype of murine TAMs *in vitro*, but only in combination with TGF-β blockade (35). We propose that exploiting the synergistic effect of combined macrophage activation with IFN-γ and TLR agonist may have a great potential for development of novel tumor immunotherapies.

## Ethics Statement

The study was approved by the Norwegian National Committee for Animal Experiments. All experiments were performed in accordance with the institutional guidelines and regulations, including EU directive 2010/63/EU.

## Author Contributions

EM performed most experimental work, analyzed results, and wrote the manuscript. PC performed experimental work, analyzed results, and contributed to writing the final version of the manuscript. SH performed experimental work on the cell line and analyzed results. AL performed early, preliminary experimental work. KB provided help with cell culture work and materials. MS provided BMDM material and protocol and contributed to writing the final version of the manuscript. IØ provided supervision and experimental help, discussed the results, and contributed to writing the final version of the manuscript. AC designed, supervised, and evaluated the experiments and contributed to writing the manuscript. All authors read and approved the final version of the manuscript.

## Conflict of Interest Statement

The authors declare that the research was conducted in the absence of any commercial or financial relationships that could be construed as a potential conflict of interest.

## Abbreviations

BMDM, bone marrow derived macrophage; cpm, counts per minute; DETA/NO, diethylenetriamine/NO adduct; FBS, fetal bovine serum; FLA, flagellin; IFN-γ, interferon-γ; iNOS, inducible nitric oxide synthase; LLC, Lewis lung carcinoma; LPS, lipopolysaccharide; LTA, lipotechoic acid; MAF, macrophage-activating factor; MIG, monokine-induced by IFN-γ; NFκB, nuclear factor kappa-light-chain-enhancer of activated B cells; NO, nitric oxide; Pam3, Pam3CSK4; Poly(I:C), polyinosinic:polycytidylic acid; SMT, s-methylisothiourea hemisulfate salt; TLR, toll-like receptor; TAM, tumor-associated macrophage.
